# RETRACTION: Six2 Promotes Non–Small Cell Lung Cancer Cell Stemness Via Transcriptionally and Epigenetically Regulating E‐Cadherin

**DOI:** 10.1111/cpr.13682

**Published:** 2024-05-28

## Abstract

H. Hou, X. Yu, P. Cong, Y. Zhou, Y. Xu, Y. Jiang, “Six2 Promotes Non–Small Cell Lung Cancer Cell Stemness Via Transcriptionally and Epigenetically Regulating E‐Cadherin,” *Cell Proliferation* 52, no. 4 (2019): e12617, https://doi.org/10.1111/cpr.12617

The above article, published online on 22 April 2019 in Wiley Online Library (wileyonlinelibrary.com), has been retracted by agreement between the Deputy Editor‐in‐Chief, Yunfeng Lin, and John Wiley and Sons Ltd. The retraction has been agreed following the authors' request to retract the article due to unreliable results and a lack of original data. Further investigation revealed multiple images previously published elsewhere in a different scientific context. Thus, the editors consider the conclusions of this manuscript substantially compromised. The corresponding author Yuhua Jiang agrees with this decision on behalf of all authors.

H. Hou, X. Yu, P. Cong, Y. Zhou, Y. Xu, Y. Jiang, “Six2 Promotes Non–Small Cell Lung Cancer Cell Stemness Via Transcriptionally and Epigenetically Regulating E‐Cadherin,” *Cell Proliferation* 52, no. 4 (2019): e12617, https://doi.org/10.1111/cpr.12617

The above article, published online on 22 April 2019 in Wiley Online Library (wileyonlinelibrary.com), has been retracted by agreement between the Deputy Editor‐in‐Chief, Yunfeng Lin, and John Wiley and Sons Ltd. The retraction has been agreed following the authors' request to retract the article due to unreliable results and a lack of original data. Further investigation revealed multiple images previously published elsewhere in a different scientific context. Thus, the editors consider the conclusions of this manuscript substantially compromised. The corresponding author Yuhua Jiang agrees with this decision on behalf of all authors.

